# P-601. Safety and Immunogenicity of a Respiratory Syncytial Virus and Human Metapneumovirus Virus-like Particle Protein Subunit Combination Vaccine in 60–85-Year-Old Adults: Interim Results from a Phase 2a Clinical Trial

**DOI:** 10.1093/ofid/ofae631.799

**Published:** 2025-01-29

**Authors:** Matthew Davis, Craig Shapiro, Mark D Adams, Max Ciarlet, Elizabeth M Adams, Nicholas Hourguettes, Judy Wen, Wasima Rida, Jennifer Price, Lee-Jah Chang, Niranjan Kanesa-Thasan

**Affiliations:** Rochester Clinical Research, Rochester, New York; Cenexel Clinical Research, Salt Lake City, Utah; AMR, Lexington, Kentucky; Icosavax, AstraZeneca, Seattle, Washington; Icosavax, AstraZeneca, Seattle, Washington; Icosavax, Inc, Seattle, Washington; Icosavax, AstraZeneca, Seattle, Washington; Icosavax, AstraZeneca, Seattle, Washington; Icosavax, AstraZeneca, Seattle, Washington; AstraZeneca, Gaithersburg, Maryland; Icosavax, AstraZeneca, Seattle, Washington

## Abstract

**Background:**

Respiratory syncytial virus (RSV) and human metapneumovirus (hMPV) often cause serious lower respiratory tract infections in older adults. In this phase 2a trial, we evaluate the safety and immunogenicity of an RSV/hMPV virus-like particle (VLP) subunit combination vaccine, IVX-A12, ± adjuvant in 60–85-year-olds (NCT05903183). We present interim data to Day 180 post vaccination.

**Figure.**
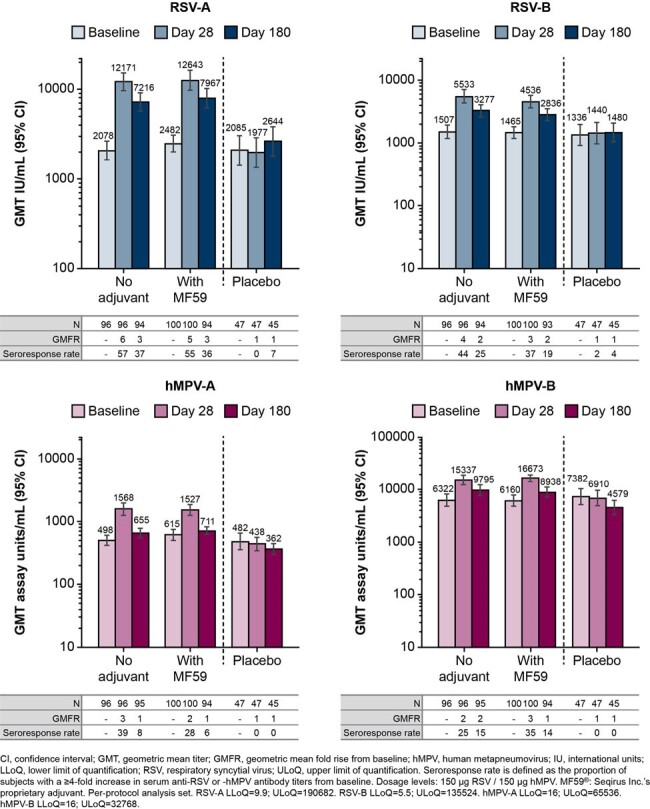
Geometric mean titers (GMTs) against RSV-A, RSV-B, hMPV-A, and hMPV-B at baseline and Days 28 and 180 post vaccination

**Methods:**

Participants were randomized 2:2:1 to receive a single dose of IVX-A12 containing 150µg RSV/150µg hMPV ± MF59® (CSL Seqirus) oil-in-water adjuvant, or placebo. Safety assessments included solicited and unsolicited adverse events (AEs), serious AEs (SAEs), AEs of special interest (AESIs), and medically attended AEs (MAAEs). Geometric mean titers (GMTs) of RSV and hMPV neutralizing antibodies are reported for Days 28 and 180.

**Results:**

In total, 264 participants were included (IVX-A12 n=103; IVX-A12+MF59 n=108; placebo n=53), with a median age of 69.2 years (range 60–85) and 57.6% were female. Solicited and unsolicited AEs were mostly mild with no related SAEs, AESIs, or MAAEs. For IVX-A12, GMTs (international units/mL) against RSV-A and B were 2078 (95% CI: 1628, 2653) and 1507 (1180, 1926) at baseline, 12171 (9680, 15303) and 5533 (4360, 7020) at Day 28, and 7216 (5706, 9124) and 3277 (2627, 4089) at Day 180, respectively (**Figure**). For IVX-A12+MF59, GMTs were 2482 (2003, 3075) and 1465 (1175, 1827) at baseline, 12643 (9829, 16263) and 4536 (3623, 5680) at Day 28, and 7967 (6219, 10206) and 2836 (2281, 3526) at Day 180, respectively.

For IVX-A12, GMTs (assay units/mL) against hMPV-A and B were 498 (412, 601) and 6322 (4841, 8255) at baseline, 1568 (95% CI 1242, 1980) and 15337 (12371, 19013) at Day 28, and 655 (550, 781) and 9795 (7788, 12319) at Day 180, respectively. For IVX-A12+MF59, GMTs were 615 (501, 754) and 6160 (4828, 7859) at baseline, 1527 (1250, 1864) and 16673 (14284, 19461) at Day 28, and 711 (614, 823) and 8938 (7128, 11209) at Day 180.

**Conclusion:**

IVX-A12 containing 150µg RSV/150µg hMPV was well tolerated and immunogenic against RSV and hMPV to 6 months in older adults up to 85 years of age, regardless of adjuvant. These data support the ongoing clinical development of an unadjuvanted RSV/hMPV VLP combination vaccine.

**Disclosures:**

**Max Ciarlet, PhD**, Icosavax/AstraZeneca: Employee of Icosavax, a member of the AstraZeneca Group, and may or may not hold stocks in AstraZeneca **Elizabeth M Adams, MD**, Icosavax/AstraZeneca: Employee of Icosavax, a member of the AstraZeneca Group, and may or may not hold stocks in AstraZeneca **Nicholas Hourguettes, BA**, Icosavax/AstraZeneca: Employee of Icosavax, a member of the AstraZeneca Group, and may or may not hold stocks in AstraZeneca **Judy Wen, BA Econ**, Icosavax/AstraZeneca: Employee of Icosavax, a member of the AstraZeneca Group, and may or may not hold stocks in AstraZeneca **Wasima Rida, PhD**, COH NCI grant for CMV vaccination of HCT-D: Grant/Research Support|Icosavax/AstraZeneca: Employee of Icosavax, a member of the AstraZeneca Group, and may or may not hold stocks in AstraZeneca **Jennifer Price, BS**, Icosavax/AstraZeneca: Employee of Icosavax, a member of the AstraZeneca Group, and may or may not hold stocks in AstraZeneca **Lee-Jah Chang, MD**, AstraZeneca: Employee of AstraZeneca **Niranjan Kanesa-Thasan, MD**, Icosavax/AstraZeneca: Previous employee of Icosavax, a member of the AstraZeneca Group

